# Mda-9/Syntenin Is Expressed in Uveal Melanoma and Correlates with Metastatic Progression

**DOI:** 10.1371/journal.pone.0029989

**Published:** 2012-01-13

**Authors:** Rosaria Gangemi, Valentina Mirisola, Gaia Barisione, Marina Fabbi, Antonella Brizzolara, Francesco Lanza, Carlo Mosci, Sandra Salvi, Marina Gualco, Mauro Truini, Giovanna Angelini, Simona Boccardo, Michele Cilli, Irma Airoldi, Paola Queirolo, Martine J. Jager, Antonio Daga, Ulrich Pfeffer, Silvano Ferrini

**Affiliations:** 1 Immunological Therapy Laboratory, National Cancer Research Institute, University Hospital San Martino, Genoa, Italy; 2 Laboratory of Integrated Molecular Pathology, National Cancer Research Institute, University Hospital San Martino, Genoa, Italy; 3 Department of Pathology, National Cancer Research Institute, University Hospital San Martino, Genoa, Italy; 4 Animal Model Facility, National Cancer Research Institute, University Hospital San Martino, Genoa, Italy; 5 Medical Oncology, National Cancer Research Institute, University Hospital San Martino, Genoa, Italy; 6 Gene Transfer Laboratory, National Cancer Research Institute, University Hospital San Martino, Genoa, Italy; 7 Ophthalmic Oncology Center, E.O. Galliera Hospital, Genoa, Italy; 8 AIRC Laboratory of Immunology and Tumors, Department of Experimental and Laboratory Medicine, G. Gaslini Institute, Genova, Italy; 9 Departments of Ophthalmology, Leiden University Medical Center (LUMC), Leiden, The Netherlands; Faculdade de Medicina, Universidade de São Paulo, Brazil

## Abstract

Uveal melanoma is an aggressive cancer that metastasizes to the liver in about half of the patients, with a high lethality rate. Identification of patients at high risk of metastases may provide indication for a frequent follow-up for early detection of metastases and treatment. The analysis of the gene expression profiles of primary human uveal melanomas showed high expression of *SDCBP* gene (encoding for syndecan-binding protein-1 or mda-9/syntenin), which appeared higher in patients with recurrence, whereas expression of syndecans was lower and unrelated to progression. Moreover, we found that high expression of *SDCBP* gene was related to metastatic progression in two additional independent datasets of uveal melanoma patients. More importantly, immunohistochemistry showed that high expression of mda-9/syntenin protein in primary tumors was significantly related to metastatic recurrence in our cohort of patients. Mda-9/syntenin expression was confirmed by RT-PCR, immunofluorescence and immunohistochemistry in cultured uveal melanoma cells or primary tumors. Interestingly, mda-9/syntenin showed both cytoplasmic and nuclear localization in cell lines and in a fraction of patients, suggesting its possible involvement in nuclear functions. A pseudo-metastatic model of uveal melanoma to the liver was developed in NOD/SCID/IL2Rγ null mice and the study of mda-9/syntenin expression in primary and metastatic lesions revealed higher mda-9/syntenin in metastases. The inhibition of *SDCBP* expression by siRNA impaired the ability of uveal melanoma cells to migrate in a wound–healing assay. Moreover, silencing of *SDCBP* in mda-9/syntenin-high uveal melanoma cells inhibited the hepatocyte growth factor (HGF)-triggered invasion of matrigel membranes and inhibited the activation of FAK, AKT and Src. Conversely syntenin overexpression in mda-9/syntenin-low uveal melanoma cells mediated opposite effects. These results suggest that mda-9/syntenin is involved in uveal melanoma progression and that it warrants further investigation as a candidate molecular marker of metastases and a potential therapeutic target.

## Introduction

Uveal melanoma is the most common primary intraocular tumor in adults with an incidence rate of about 7 new cases per one million individuals each year [1], [2] About 50% of patients develop metastases, mostly to the liver, within ten years from diagnosis and their median survival is 5 to 7 months after detection of metastatic lesions. The specific tropism of metastatic spreading, together with the existence of specific genetic and molecular markers of uveal melanoma, indicate that it is a distinct tumor from the more common cutaneous melanoma [3]. Despite the improvements in diagnosis and the development of more effective local therapies for primary tumors, the rate of metastatic death remains unchanged. Metastases are usually detected 2 to 5 year after ocular treatment and are frequently resistant to chemotherapy [4]. Therefore it seems important to identify high-risk patients at the time of the initial diagnosis for early detection and treatment of metastatic disease or for the administration of adjuvant therapy. A great effort has been made to understand the biological mechanisms involved in the spreading and growth of uveal melanoma metastases and to discover new prognostic markers. Hepatocyte growth factor/scatter factor (HGF) [5]–[7], Insulin-like growth [8] and Stem cell factor [9]–[11] receptors have been involved in metastatic progression of uveal melanoma. In addition, the chemokine receptor CXCR4 was recently related to liver homing of human uveal melanoma metastatic cells [12]. However, the mechanisms responsible for hematogenous tumor dissemination and liver localization of metastases are still poorly understood. A classification of uveal melanoma patients in two classes with different prognosis (class-1 low-risk and class-2 high risk) has been proposed on the basis of a specific tumor molecular signature identified by gene expression profiling. [13], [14]. As few as three genes (*PHLDA1, FZD6 and ENPP2*) correctly predicted the class of all tumor samples. Inhibitor of DNA binding 2, *ID2*, down regulated in class-2 (i.e. high-risk) tumors, was later reported by the same authors as the top class discriminating gene [14], the loss of which causes an increase in the rate of liver metastasis in a transgenic mouse model of ocular melanoma [15]. More recently, the usage of a 15-gene PCR-based assay has been proposed as a suitable method for the routine identification of the high-risk phenotype [16].

In the search of possible molecular pathways involved in uveal melanoma progression we focused our attention on *SDCBP* gene, which we detected as a highly expressed gene through a microarray analysis. *SDCBP* gene encodes for the syndecan-binding protein syntenin-1, also known as melanoma differentiation associated gene 9 (mda-9) [17]. Mda-9/syntenin is a scaffolding-PDZ domain-containing protein with multiple biological activities [18], [19]. These include syndecan binding and recycling [20], [21] clustering of membrane receptors [22], intracellular trafficking [23], Sox4 activation, and signal transduction [24]. Mda-9/syntenin is able to influence the cell shape and also the migration and invasion ability of different types of cancer cells, including cutaneous melanoma [25]–[29] where high *SDCBP* expression has been related to metastatic spreading [25].

In the present work we find an elevated expression of *SDCBP* by gene expression profiling in a cohort of 29 primary uveal melanomas. Moreover, high levels of mda-9/syntenin protein are present in uveal melanoma cell lines, primary cultures and biopsies of human primary tumors. Interestingly, high expression of mda-9/syntenin protein is significantly related to metastatic progression. In an animal model of pseudo-metastasis, mda-9/syntenin expression is higher in metastases than in the primary site, suggesting an active role of mda-9/syntenin in metastatic spreading of uveal melanoma cells. This possibility is also supported by the finding that mda-9/syntenin is involved in cell migration of uveal melanoma cells in culture and in invasiveness and activation of focal adhesion kinase (FAK), AKT and Src triggered by HGF.

## Materials and Methods

### Cell cultures

The human uveal melanoma cell lines Mel 270, 92.1, OMM1 and OMM2.5 [30]–[31] were cultured in RPMI 1640 (Gibco-BRL, Rockville, MD, USA) supplemented with 10% fetal bovine serum (FBS), 2 mM L-glutamine and 100 U/ml penicillin/streptomycin at 37°C. Primary cultures were obtained from tumor biopsies, upon approval of the institutional bioethics board and informed written consent of the patients, by mechanical dissociation and cultured in the medium above.

### Tumor samples

Tissue samples were obtained from 29 uveal melanomas after enucleation surgery upon approval of the institutional bioethics board and informed written consent of the patients. Samples for gene expression profiling were removed within 15 minutes after surgery and conserved in RNAlater (Ambion, Monza, Italy) at −20°C until processing. Mean age of the patients (12 women and 17 males) was 64 (range 52–78). DNA copy number data were available for 14 of these patients and the chromosome 3 monosomy was inferred in the other patients from gene expression profile data through a gene expression based classifier for monosomy of chromosome 3. For the 14 tumor samples for which DNA copy number alteration and gene expression profile data were available we identified the genes whose expression levels were related to chromosome 3 status. We calculated the centroids for monosomic and disomic samples and classified the remaining 15 samples, for which no copy number alteration data were available, according to the distance from the centroid of each class (manuscript in preparation). Chromosome 8 polysomy was evaluated by DNA copy number in 14 patients and by FISH in the other patients.

### Gene expression profile analysis

Tumor samples were homogenized in the tissue lyser Mixer Mill (Qiagen, Hilden, Germany) in total RNA extraction lysis buffer using RNeasy (Qiagen). RNA quality was assessed in the BioAnalyzer (Agilent, St. Clara, CA). RNA Integrity Number (RIN) was evaluated and only samples with RIN>or = 7 were considered acceptable. cDNA synthesis was performed using T7-(dT)24 oligo primers and the Custom SuperScript Double-Stranded cDNA Synthesis Kit (Invitrogen, Irvine, CA, USA). Double stranded cDNAs were extracted with phenol-chloroform-isoamyl alcohol (25∶24∶1), ethanol precipitated, and used to prepare cRNAs using the Bioarray High Yield RNA Transcription Kit (Affymetrix, Santa Clara, CA, USA) according to the manufacturer's instructions. cRNAs were purified using the RNeasy Mini Kit (Qiagen), controlled by agarose gel electrophoresis and subjected to fragmentation for 35 min. at 94°C in fragmentation buffer (40 mM Tris-acetate pH 8.1, 100 mM CH3COOH, 30 mM Mg(CH3COO)2×4H2O).

GeneChip microarray analysis and data normalization - Labeled cRNA was used for screenings of GeneChip Human Genome U133plus2 arrays (Affymetrix, Santa Clara, CA, USA). Hybridization and scanning was performed on the Affymetrix platform [32]. Data were preprocessed following the RMA procedure of Bioconductor 2.8 using quantile normalization (http://www.bioconductor.org). All microarray data is MIAME compliant. The dataset, corresponding to 29 uveal melanoma primary tumors is available from the GEO database (http://www.ncbi.nlm.nih.gov/geo/), under accession number GSE27831.

### RT-PCR analysis

cDNA was synthesized with oligo dT primers from 2 micrograms of total RNA with Superscript II (RT Invitrogen). For conventional RT-PCR two microliters of cDNA were amplified with 2.5 IU of Taq Polymerase (Roche), by using the following primers: Human *SDCBP* upper primer: 5′TGG TGG CTC CTG TAA CTG GTA A, lower primer: 3′TGC ATG GTA ATC GTC CGT TCA A. Human *ACTB* upper primer: 5′GTG GGG CGC CCC AGG GGC ACC A, lower primer: 5′CTC CTT AAT GTC ACG CAC GAT TTC.

qPCR was performed on LightCycler480 II (Roche Applied Science) using 10 µl of LightCycler 480 SYBR Green I Master (Roche Applied Science), 2 µl of cDNA (5×diluted), 0.3 µmol sense and antisense primers in a final reaction volume of 20 µl. After amplification, melting curves with 65 steps of 15 s and 0.5°C increase were performed. Expression data were normalized on the mean of *GAPDH* gene expression data. Relative expression values were obtained using Qgene software. *GAPDH* upper primer: GAA GGT GAA GGT CGG AGT, lower primer: CAT GGG TGG AAT CAT ATT GGA A.

### Immunostaining

Immunostaining of cultured cells or frozen sections of uveal melanoma tumors derived patients and mice was performed using the rabbit anti-mda-9/syntenin antibody ab19903 (1∶100Abcam, UK) [33]. To detect mda-9/syntenin, cells were fixed for 15′ with 4% paraformaldehyde (PFA) and incubated with the appropriate antibody in PBS, 0.3% Triton X100 and 10% FBS. Binding of primary antibodies was revealed with appropriate secondary DyLight 488 affiniPure Goat Anti-Rabbit IgG (H+L) Jackson Immunoresearch laboratories, USA. Nuclei were stained by 5 minutes incubation in Propidium Iodide solution (100 ng/ml). To determine cytoplasmic and/or nuclear localization of mda-9/syntenin slides were examined using a laser-scanning FV500 microscope equipped with 488, 543 and 633 nm lasers, and coupled to an inverted IX81 platform (all from Olympus Optical, Tokio, Japan). Digital images were acquired with Fluoview 4.3b software.

### Immunoblot analysis

Cells were lysed in 20 mM Tris-HCl, pH 7.5, 150 mM NaCl, 1 mM EDTA, 1% NP40) containing protease inhibitors (Complete C Mini, Roche Applied Science, Indianapolis, IN) and 1 mM sodium orthovanadate. Nuclear extracts were prepared with Nuclear Extract Kit (ACTIVE MOTIF) USA, following manufacturer's instructions. Cell lysates were then quantified using the Bio-Rad Protein Assay (Bio-Rad, Hercules, CA). A standard Western blot analysis was then performed. Briefly, 50–100 µg of each sample were run on 10% polyacrylamide gel. Gel was then blotted to nitrocellulose membranes (Hybond-C Extra, Amersham GE Healthcare, Little Chalfont, UK) according to standard procedures and stained with antibodies to mda-9/syntenin, HDAC1, Actin (Sigma-Aldrich), anti-FAK(pY^397^), anti-Src (pY418) and anti-Src pan (Invitrogen), and anti-FAK (Cell Signaling), anti-phospho-AKT (Ser473), anti-AKT (pan), anti-phospho-MET (Tyr1234/1235) and anti-MET (L41G3) (Cell Signaling). Bands were visualized by a standard chemiluminescence method (Amersham ECL Plus, GE Healthcare, Little Chalfont, UK). Band intensity was evaluated by densitometric analysis with the KODAK ID image analysis software (Kodak, Rochester, NY). Intensity of phosphorylated bands was normalized to the correspondent unphosphorylated band.

### FACS analysis

Cell were stained after permeabilization with anti-mda-9/syntenin antibody and anti-c-MET (Santa Cruz Biotechnology, Santa Cruz, CA, USA), washed and incubated with DyLight 488 affiniPure Goat anti-rabbit secondary antibody. Cell fluorescence was analyzed in a FACScan (Becton&Dickens).

### Immunohistochemistry

Immunohistochemical (IHC) detection of mda-9/syntenin was performed on formalin-fixed, paraffin-embedded sections from 29 enucleated primary uveal melanomas with known clinical history (Table 1) and on three liver metastases. For each sample the more representative tumor sections (3 µm) were immunostained using a BenchMark XT automated stainer (Ventana Medical Systems, SA Strasbourg France). The sections were deparaffinized and antigen-retrieval was performed with high pH citrate buffer. The primary antibody was used at 1∶200 dilution of a commercially available anti-mda-9/syntenin rabbit polyclonal antiserum (Abcam, Cambridge) for 30 min at 37°C. The antibody complex was revealed with the Polymeric System (Ultraview Red Ventana Medical System/Alkaline phosphatase) and the Amplification Kit (Ventana Medical System). Then the sections were counterstained with modified Gill's hematoxylin and mounted in Eukitt (Bio-Optica, Milano, Italy). An appropriate positive tissue control was used for each staining run; the negative control consisted of immunohistochemistry procedure on adjacent sections in the absence of the primary antibody. The sections were observed with an Olympus light microscope using ×10, ×40 and ×63 objectives. The immunostaining was independently evaluated by two experienced pathologists using a previously reported semi-quantitative scoring system [34]. The extent of positively labeled cells was ranked into 5 grades, i.e. 0 = 0%, 1 = 1–10%, 2 = 11–50%; 3 = 51–90% and 4 = >90%; staining intensity was graded into 4 steps with 0 = no staining; 1 = low; 2 = moderate and 3 = strong staining. Results were presented as product of the two assessments thus ranging from 0 to 12. Patient samples were then classified into two categories that expressed mda-9/syntenin higher or lower than the median value.

**Table 1:** Clinicopathological characteristics of patient samples and expression of mda-9/syntenin by immunohistochemistry.

Specim. n	Gender	Age at proced.	Thickn. (mm)	Largest diamet. (mm)	Location	Cell type	Sclera	Met	DFS months	Chr 3	Chr 8	Previous Treatment	mda-9/syntenin
MU_1	m	78	7	6	anterior	spindle	y	n	67	d	p	none	L
MU_10	m	84	14	25	anterior	mixed	y	y	21	m	d	none	H
MU_11	f	61	10	14	middle	mixed	n	n	55	m	d	proton beam	L
MU_12	f	74	5	10	middle	mixed	nd	n	41	m	p	proton beam	H
MU_13	m	70	16	17	middle	epithelioid	n	n	55	d	d	none	H
MU_15	m	74	6	13	posterior	epithelioid	n	y	33	m	p	proton beam	H
MU_17	m	60	9	11	posterior	epithelioid	n	n	52	d	d	none	L
MU_2	f	82	3	2	anterior	spindle	y	n	20	m	p	proton beam	L
MU_22	m	74	7	17	middle	NP	n	n	40	d	d	proton beam	H
MU_25	m	33	7,56	15	middle	spindle	y	n	15	d	d	none	L
MU_3	m	64	15	23	middle	mixed	y	y	31	m	d	none	H
MU_30	f	65	NP	15	middle	NP	y	n	36	m	na	none	H
MU_31	f	69	4,9	16	posterior	mixed	y	y	17	m	p	none	H
MU_32	f	76	5,3	12	posterior	mixed	y	n	44	d	d	none	L
MU_33	m	59	5,2	14	middle	spindle	y	n	43	m	d	none	L
MU_34	m	51	10,5	15	middle	mixed	y	y	19	m	p	none	H
MU_36	m	80	16	12	middle	epithelioid	n	y	19	m	p	none	L
MU_4	m	61	6	11	posterior	spindle	n	y	31	m	d	none	H
MU_40	m	51	NP	16	NP	epithelioid	y	n	40	m	d	none	L
MU_5	m	85	12	16	middle	spindle	y	n	48	d	d	none	H
MU_6	m	48	8	12	middle	mixed	n	n	57	d	d	none	L
MU_7	f	69	13	12	anterior	epithelioid	n	y	25	m	p	none	H
MU_8	f	74	7	9	middle	mixed	n	y	18	d	d	none	H
MU_9.1	m	66	6	14	middle	mixed	n	y	17	m	p	none	L
MU16	f	77	7	9	posterior	mixed	n	n	54	d	d	none	L
MU18	m	62	4	20	posterior	spindle	y	n	41	m	d	none	L
MU20	f	55	11	13	middle	spindle	n	n	48	d	d	none	L
MU21	f	42	10	12	posterior	mixed	n	y	51	m	p	none	H
MU23	f	71	NP	16	NP	spindle	y	n	42	m	d	Protonbeam	L

Met: metastasis; Chr: chromosome; DFS: disease free survival. nd: not done; NP: not provided. In the eleventh column m: monosomy; d: disomy. In the twelft column p: polisomy; d: disomy. In the thirteenth column: previous patients treatment. In the fourteenth column: mda-9/syntenin expression level, L: low expression; H: high expression.

### Survival and statistical analysis

Disease-free survival curves were constructed by using the Kaplan-Meier method and the Wilcoxon log-rank test was used to compare the curves. Disease Free Survival (DFS) was defined as the elapsed interval from date of eye removal or biopsy to date of last follow-up or melanoma-related metastasis. Statistical analysis was performed using Prism 5 (Graph-Pad Software, San Diego, Ca, USA). All other data were compared using the Student's t-test, and a p-value<0.05 was considered statistically significant.

Small Interfering RNA (siRNA) transfection and wound-healing and invasion assays. 

ON-TARGET plus SMART pool for human *SDCBP*or siCONTROL Non-Targeting siRNA pool (Dharmacon, Lafayette, CO) were transfected in 92.1 and Mel270 cells using Interferin, polyplus transfection (Invitrogen) following the the manufacturer's instructions. Efficiency of siRNA inhibition was evaluated at the protein level by western blot analysis.

Transfected cells were assayed in a wound-healing assay to assess cell motility in two dimensions. Cells were plated overnight to achieve a subconfluent cell layer in 24 well-plates. A scratch was made on the cell layer with a micropipette tip, and floating cells were removed by two washes with serum-free medium. Cells were then incubated in culture medium and ‘Wound-healing’ was visualized by comparing photographs taken at the time of scratching and 24 hours later, by a Nikon DS-5M Camera System mounted on a phase-contrast Leitz microscope. The distance migrated by the cells was determined by measuring the wound width after 24 hours, and subtracting it from the wound width at time 0. Three experiments were performed in quadruplicates.

BD BioCoat invasion chambers coated with growth factor reduced Matrigel were purchased from BD Biosciences (Sparks, MD) for invasion assays. One hundreds thousand cells diluted in 0.5 ml of medium containing 0.1% FCS were added to the top chambers of 24-well transwell plates (BD Biosciences; 8- µm pore size), and assay media, with or without 100 ng/ml of recombinant HGF (Peprotech) or 10% FCS or 50% MG63 supernatant were added to the bottom chambers. HGF-containing conditioned medium of the MG63 cell line was kindly provided by Dr. Daniela de Totero, IST Genova. After 48 hours incubation, top (nonmigrated) cells were removed, and bottom (migrated) cells were fixed and stained with 1% toluidine blue to visualize nuclei. The number of migrating cells in five fields were counted under ×200 magnification, and the mean for each chamber determined. Experiments were run in triplicate.

### Gain of function experiments

The full length human *SDCBP* cDNA fragment was amplified by RT-PCR from 92.1 cells and cloned into eukaryotic expression vector pIRESneo (Clontech). Correct nucleotide sequence of *SDCBP*-cDNA was confirmed by sequencing (BigDye® Terminator v3.1 Cycle Sequencing Kit, Applied Biosystem and 3130XL, Life Technology). Recombinant plasmid pIRESneo-*SDCBP* or empty vector were transfected into Mel 270 cells by Lipofectamine 2000 reagent (Invitrogen). After 48 hours transfected cells were lysed and cytoplasmic extracts were analyzed on western blots.

### In vivo experiments

The animals were housed in pathogen-free conditions. The experiments were performed according to the National Regulation on Animal Research Resources and approved by the Istitutional Review Board for animal experimentation (Approval ID Number: IST 284). All mice used in the study were anesthetized with intra-peritoneal injections of ketamine and xylazine. For the induction of liver metastases, 92.1-luciferase gene-transduced (luc) or Mel 270-luc melanoma cells (10^7^–10^6^ cells in 100 µl) were implanted under the spleen capsule of six NU/NU mice (4–8 weeks old from Janvier, France), and nine NOD/SCIDIL2Rγ null mice (Jackson Laboratory (Bar Harbor, ME). Mice were inspected at weekly intervals by the IVIS (IVIS imaging 100, Xenogen, Caliper LifeSciences, France), after intraperitoneal injection of 150 mg/kg of luciferine, Promega Italia, Milano. Mice underwent necropsy 9 to 43 days later, when tumor outgrowth into the liver was clearly evident. Part of the livers and spleens were frozen in liquid nitrogen for immunohistochemical studies. Macroscopic metastatic foci and primary tumors were also aseptically isolated and cultured in complete medium for short term to evaluate mda-9/syntenin expression by immunofluorescence.

## Results

### High level of SDCBP expression in primary uveal melanomas and cell lines

**Figure 1:**
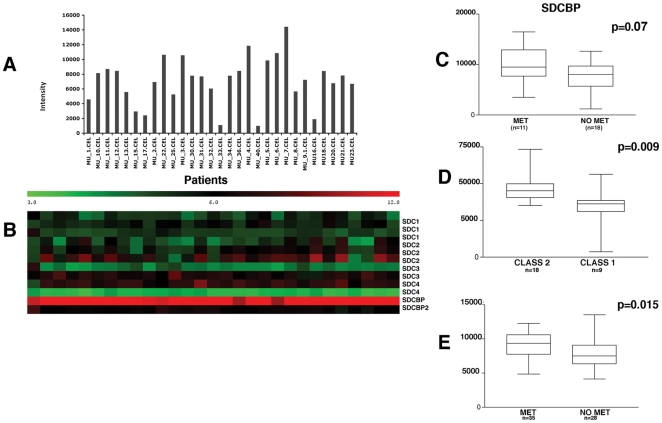
Gene expression profile of primary uveal melanomas reveals high but heterogeneous expression of *SDCBP*. **A:** Bars indicate intensitiy of *SDCBP* expression in 29 primary uveal melanoma analyzed by gene expression profiling in the present study. **B:** Heat map showing the expression levels of syndecan (*SDC*)-1, -2, 3 and -4 genes, *SDCBP* and syntenin-2 (*SDCBP2*). **C:** Comparrison of *SDCBP* expression in metastatic and non-metastatic patients (n = 29) in our cohort showed a trend to higher *SDCBP* expression in metastatic patients (p = 0.07). The same type of comparison performed on gene expression profile data from Onken et al. (D), between class1 (low-risk) and class 2 (high risk) patients (n = 27) [13] and on gene expression profile data from Laurent et al. (E) between metastatic and non-metastatic patients (n = 63) [35] showed significantly elevated levels of *SDCBP* in high risk and metastic patients, respectively.

In the search for new potential molecular pathways of progression, we have performed gene expression profiling using high-density microarrays of 29 primary uveal melanomas. *SDCBP* was highly expressed in all the samples studied, though at variable levels (Fig. 1 A). Differently, the genes of syndecans (*SDC 1*, *2*, *3* and *4*) were expressed at relatively low levels, although *SDC2* showed higher expression than the other members of the same family in nine patients (Fig. 1B). Notably, syndecan binding protein-2 (*SDCBP2*) was not expressed in any of the samples tested (Fig. 1B).

Eleven patients out of 29 developed liver metastases during follow-up (on average after 36.7 months, range 15 to 67 months). High *SDCBP* mRNA expression conferred a risk with Odds Ratio of 9.0 (p = 0.01, IC 95% 1.46–55.48) for recurrence, which was as strong as monosomy 3 (OR: 12.50, p = 0.01, IC 95% 1.31–119.33), in our cohort. Both monosomy 3 and *SDCBP* overexpression significantly correlated with the occurrence of metastasis (monosomy r = 0.46 p = 0.01, *SDCBP* r = 0.47, p = 0.001). The differential expression of *SDCBP* between metastatic and non-metastatic patients (median 9484 as compared to 8330 as average of normalized intensity values) showed a trend to statistical significance (P = 0.07) in our cohort (Fig. 1 C). It must be emphasized that our cohort had a rather short follow-up (maximum 67 months) and that the number of metastatic patients was lower (37.9%) than that expected (50%) at a longer follow-up, possibly limiting the achievement of statistical significance.

Therefore, to further evaluate the possible correlation of *SDCBP* expression with high metastatic risk, we analyzed the expression profile of another cohort of 27 patients with a longer follow-up, kindly provided by Dr Onken. A molecular signature predictive of metastatic progression and death, was previously identified in this cohort by the authors [13]. Since *SDCBP* gene expression was not addressed in this previous study, we analyzed their raw data for *SDCBP*, with the aim to corroborate our findings on a different cohort. Expression of *SDCBP* was significantly higher (p = 0.009) in class-2 (high risk) than in class-1 (low risk) cases (Fig. 1D), according to the classification proposed by the authors. In addition, when the Onken classifier was applied to our microarray data, we found that all metastatic patients, but one, fell in the high-risk class. Moreover, *SDCBP* was found to be expressed at a significantly higher level (p = 0.015) in metastatic compared to non-metastatic patients in an additional gene expression profile dataset of 63 uveal melanoma patients (Fig. 1E), recently reported by Laurent et al [35].

**Figure 2:**
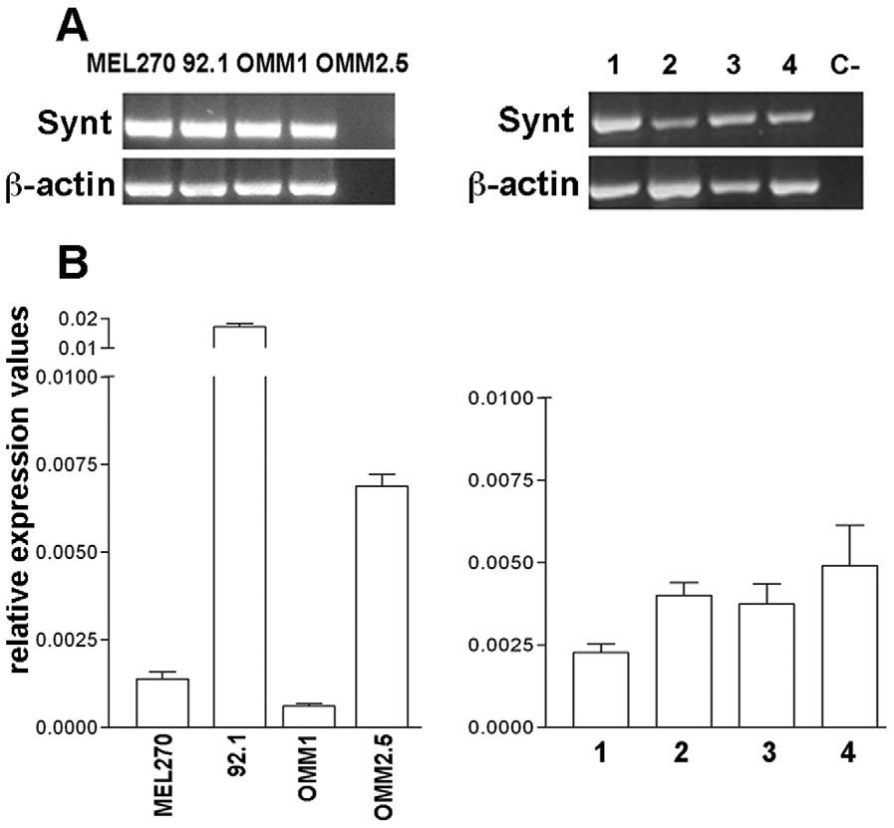
*SDCBP* mRNA is expressed in uveal melanoma cells. **A:** Conventional RT-PCR analysis of *SDCBP* gene expression in cell lines derived from primary tumors (MEL 270 and 92.1), cell lines derived from metastatic lesions (OMM1 and OMM2.5) and from four primary cultures derived from patients' ocular tumors (1,2,3,4). The lane identified by “C-” indicates negative control in the absence of cDNA. **B:** qPCR analysis of *SDCBP* mRNA expression in uveal melanoma cell lines and primary cultures. Expression values are normalized on the mean of *GAPDH* gene expression.

We further analyzed *SDCBP* expression in human uveal melanoma cell lines and primary cultures derived from four primary uveal melanomas, (fig. 2A). MEL 270 and 92.1 cell lines, which derive from primary tumors, and OMM1 and OMM2.5 deriving from skin and liver metastases, respectively [30]–[31] clearly expressed *SDCBP*, thus confirming that *SDCBP* is expressed by uveal melanoma neoplastic cells. qPCR analysis showed that *SDCBP* gene expression was higher in the 92.1 than in MEL 270 cell lines and in the OMM2.5 hepatic metastasis than in the OMM1 cutaneous metastasis cell line. Of note, the OMM2.5 metastatic cell line showed higher expression than the MEL 270 primary tumor cell line, derived from the same patient [36]. A certain degree of heterogeneity of *SDCBP* expression was also observed in primary cultures (Fig. 2B).

### Analysis of mda-9/syntenin protein expression in uveal melanoma

**Figure 3:**
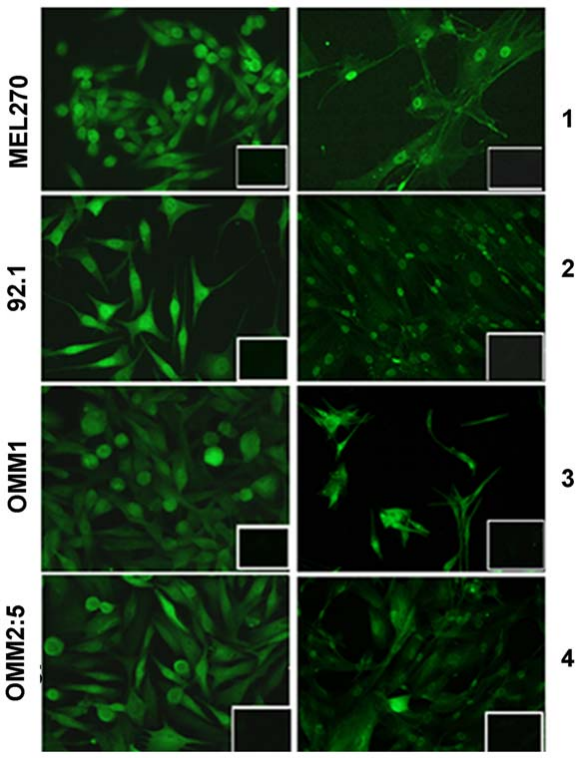
Analysis of Mda-9/syntenin protein expression in uveal melanoma cell lines. **A:** Immunostaining of fixed and permeabilized cell lines (left) and primary cultures (right). Original magnification 400×. The insets show the negative control performed by the use of non-immune rabbit Ig.

**Figure 4:**
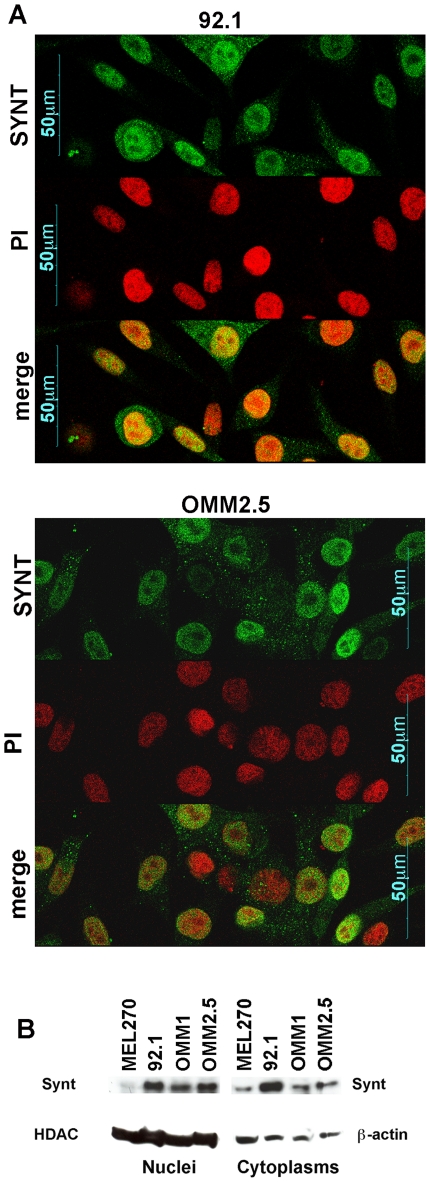
Mda-9/syntenin is expressed in the nucleus of uveal melanoma cells. **A:** Confocal fluorescence microscopy shows nuclear and cytoplasmic localization of mda-9/syntenin in 92.1 cells (upper panels) and OMM2.5 cells (lower panels). An optical section with mda-9/syntenin staining (green) and propidium iodide (red) is shown. A merging image is shown in the bottom quadrants of each panel (original magnification 600×). **B:** Western blot analysis showing nuclear and cytoplasmic expression of mda-9/syntenin. HDAC1 and β-actin were used as loading controls for nuclear and cytoplasmic extracts, respectively.

Mda-9/syntenin protein expression was first confirmed by immunofluorescence on cultured cells. Figure 3 shows that mda-9/syntenin protein is present in all the four uveal melanoma cell lines and in the four primary cultures. Interestingly, mda-9/syntenin seemed localized not only in the cytoplasm but also in the nucleus, particularly in the 92.1 and OMM2.5 cell lines. Indeed, confocal microscopy showed localization of mda-9/syntenin protein both in the cytoplasm and in the nuclei of 92.1 and OMM2.5 cells (Figure 4A and Figure S1). The nuclear and cytoplasmic expresssion of mda-9/syntenin was further confirmed by western blot analyses performed on cytoplasmic and nuclear extracts of uveal melanoma cell lines, with the strongest nuclear expression in 92.1 and OMM2.5 cells (Figure 4B). Therefore the pattern of mda-9/syntenin localization in uveal melanoma cells seemed different from the cytoplasmic and sub-membrane expression previously reported in cutaneous melanoma.

**Figure 5:**
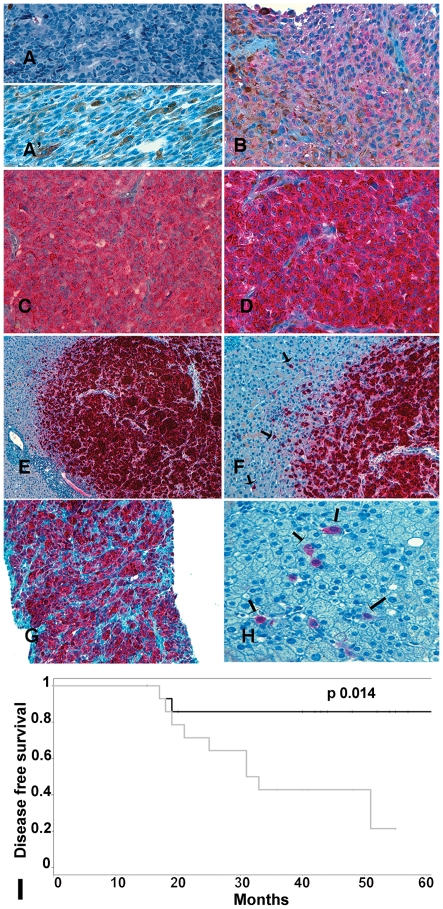
Immunohistochemical analysis of mda-9/syntenin in tissue sections of primary uveal melanomas shows correlation with metastatic progression. **A:** mda-9/syntenin expression in a choroidal metastasis of colon adenocarcinoma **A′:** primary uveal melanoma stained by secondary antibody in the absence of anti-mda-9/syntenin antibody (negative control). **B, C, D:** representative primary uveal melanomas displaying low, medium or high levels of mda-9/syntenin expression, respectively (original magnification 400×). **E:** liver metastasis of uveal melanoma (original magnification 100×). **F:** the same section at higher magnification (200×). Arrows indicate single cells of UM positive for mda-9/syntenin, which infiltrate the mda-9/syntenin-negative liver parenchima. **G:** liver metastasis of uveal melanoma from a different patient. **H:** the same at higher magnification. **I:** Kaplan-Meier analysis of Mda-9/syntenin protein expression and disease-free survival in patients with primary tumors. Patients with low mda-9/syntenin expression (dark line) showed longer survival than patients with high expression (gray line) (P<0.014). Patients were stratified according to a combination of qualitative/semi-quantitative grading. Censored patients are indicated in each curve.

Mda-9/syntenin expression was then analyzed in sections of the primary tumors from our cohort and of three metastatic livers by immunohistochemistry. All primary tumors analyzed showed staining with anti-mda-9/syntenin antibodies, although with different intensities. Tumor sections from three representative cases showed low, medium or high levels of mda-9/syntenin (Fig. 5 B, C and D, respectively). A choroidal metastasis of colon adenocarcinoma was completely negative for mda-9/syntenin (Fig. 5A), confirming the specificity of staining in uveal melanomas. Fifteen tumors were classified as high-mda-9/syntenin and fourteen as low-mda-9/syntenin on the basis of a scoring system taking into account both overall intensity and percentage of positive cells ([34] and Material and methods). Nine patients with high-mda-9/syntenin tumors developed metastatic progression, while among the fourteen patients of the mda-9/syntenin-low group only two developed metastasis. High syntenin protein expression conferred a risk with Odds Ratio of 11.70 (p = 0.005, IC 95% 1.85–74.19). In addition, Kaplan-Meier analysis showed that the high-mda-9/syntenin phenotype is significantly (p = 0.014) related to relapse (Fig. 5 panel I), suggesting its potential role as a marker of progression. Also in tumor sections, different degrees of nuclear expression, ranging from very few cells to >90% of cells, were evident (Figure S2). The nuclear score of mda-9/syntenin seemed not related to progression (p = ns by Kaplan-Meier analysis, data not shown), although the trend was towards high expression cases showing an increased risk.

The *SDCBP* gene is located on the long arm of chromosome 8 (8q12.1), which is often amplified in high-risk patients. However, no significant correlation was found between mda-9/syntenin overexpression either as mRNA or protein, and amplification of the long arm of chromosome 8. Interestingly, mda-9/syntenin protein was also very strongly expressed on three liver metastases (two of which are shown in figure 5 E–H). Staining for mda-9/syntenin allowed the detection of single cells invading the normal liver parenchyma, which was completely negative for mda-9/syntenin-1 expression (Fig. 5 F,H, arrows).

### Overexpression of mda-9/syntenin in liver metastases of uveal melanoma xenograft models

Data from gene expression profiling and immunohistochemistry suggested the hypothesis that mda-9/syntenin could be related to an invasive behavior of uveal melanoma cells. To further test this hypothesis we developed a pseudo-metastatic xenotransplant model of human uveal melanoma in immunodeficient mice. The spleen was chosen for tumor cell implantation because in this site tumor cells have access to the portal vein circulation and will have a greater likelihood of forming liver metastases. Indeed, liver metastases were detected by IVIS analysis at 40 days on average, (range 30–70) after intrasplenic inoculation of 92.1 or Mel 270 luc cells in all (6/6) nude mice. Moreover, NOD/SCIDIL2Rγ null mice (9/9) developed liver metastases detectable at IVIS analysis as soon as 20 days on average (range 14–23) after intrasplenic injection. This earlier development of metastases in the NOD/SCIDIL2Rγ null mice (Figure S3) is likely related to a more profound immune defect and to the lack of NK cells in this strain of mice [37].

**Figure 6:**
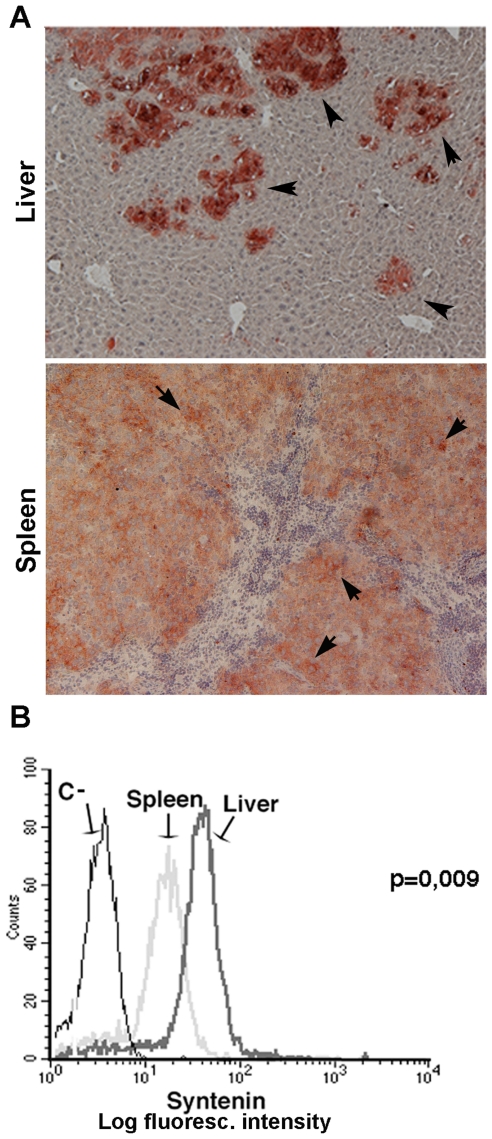
Mda-9/syntenin expression in a pseudo-metastatic model of uveal melanoma obtained by injection of human 92.1 cells under the spleen capsule of NOG mice: mda-9/syntenin expression is higher in liver metastases than in spleen. **A:** Immunohistochemistry of murine splenic uveal melanoma and liver metastases (Original Magnification 400×). Arrows indicate single cells of uveal melanoma strongly positive for mda-9/syntenin present in the spleen; arrowheads indicate mda-9/syntenin positive metastatic cells in the liver. **B:** Flow-cytometric analysis of intracellular mda-9/syntenin expression in permeabilized 92.1 cell derived from splenic tumor and liver metastases, C- is the negative control.

The expression of mda-9/syntenin was then studied in frozen sections of spleen and liver of transplanted mice by immunohistochemistry. Mda-9/syntenin immunostaining appeared moderate in spleen tumors, the primary site of implant, though single cells showed a bright staining (Fig. 6 A). Metastatic lesions, instead, stained more intensely for mda-9/syntenin (Fig. 6A) than the splenic tumor, whereas the normal hepatic tissue was negative. To confirm the differential expression of mda-9/syntenin in the tumor metastases compared to the primary tumors, tumor cells were obtained from both tumor lesions, grown in vitro for a few days and analyzed by cytofluorimetric analysis. As shown in Fig. 6B the mda-9/syntenin expression was significantly higher in the cells from liver metastases than in cells derived from the splenic tumor (mean fluorescence intensity was 1.92±0.343-fold higher in liver-derived cells than in spleen-derived cells, p = 0.0096). The expression level of the CD44 molecule was unchanged in both cell populations (data not shown).

### Silencing of SDCBP expression inhibits cell migration and invasiveness

**Figure 7:**
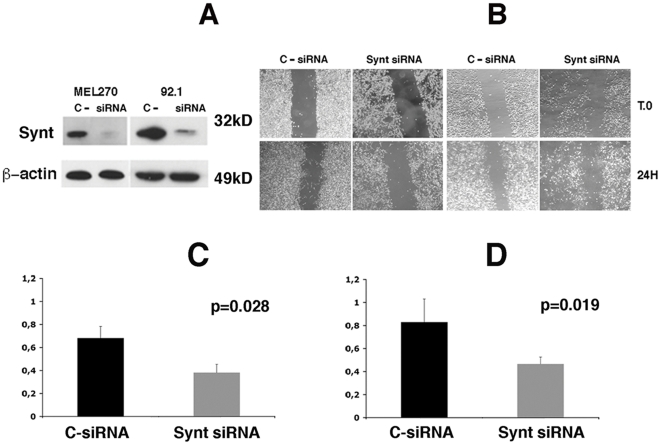
Silencing of *SDCBP* by siRNA inhibits uveal melanoma cell migration. **A:** Western blot analysis of MEL 270 and 92.1 cell lines upon 72 hrs treatment with scrambled siRNA (C), and *SDCBP* targeting siRNA (Synt). **B:** wound-healing assay on MEL 270 and 92.1 cell lines treated with scrambled siRNA (C) or with *SDCBP* targeting siRNA (Synt). Mean of migration distance of MEL 270 cells (**C**) and 92.1 (**D**) treated with scrambled siRNA (black bars) or with *SDCBP* targeting siRNA (grey bars), P values are indicated.

To assess the possible role of mda-9/syntenin in uveal melanoma metastatic process we silenced *SDCBP* expression by siRNA in 92.1 and Mel 270 cells with siRNA and studied the effects on cell migration. Western blot of the two cell lines, treated with *SDCBP* targeting siRNA, demonstrated over 80% reduction of the mda-9/syntenin protein expression compared to the cells treated with scrambled siRNA (Fig. 7A). As shown in Fig. 7B, the inhibition of mda-9/syntenin expression in Mel 270 and 92.1 cells impaired their ability to migrate in a wound–healing assay. The inhibition of migration was statistically significant in both Mel 270 (p = 0.028) and 92.1 (0.019) cells (Fig. 7, C and D respectively).

**Figure 8:**
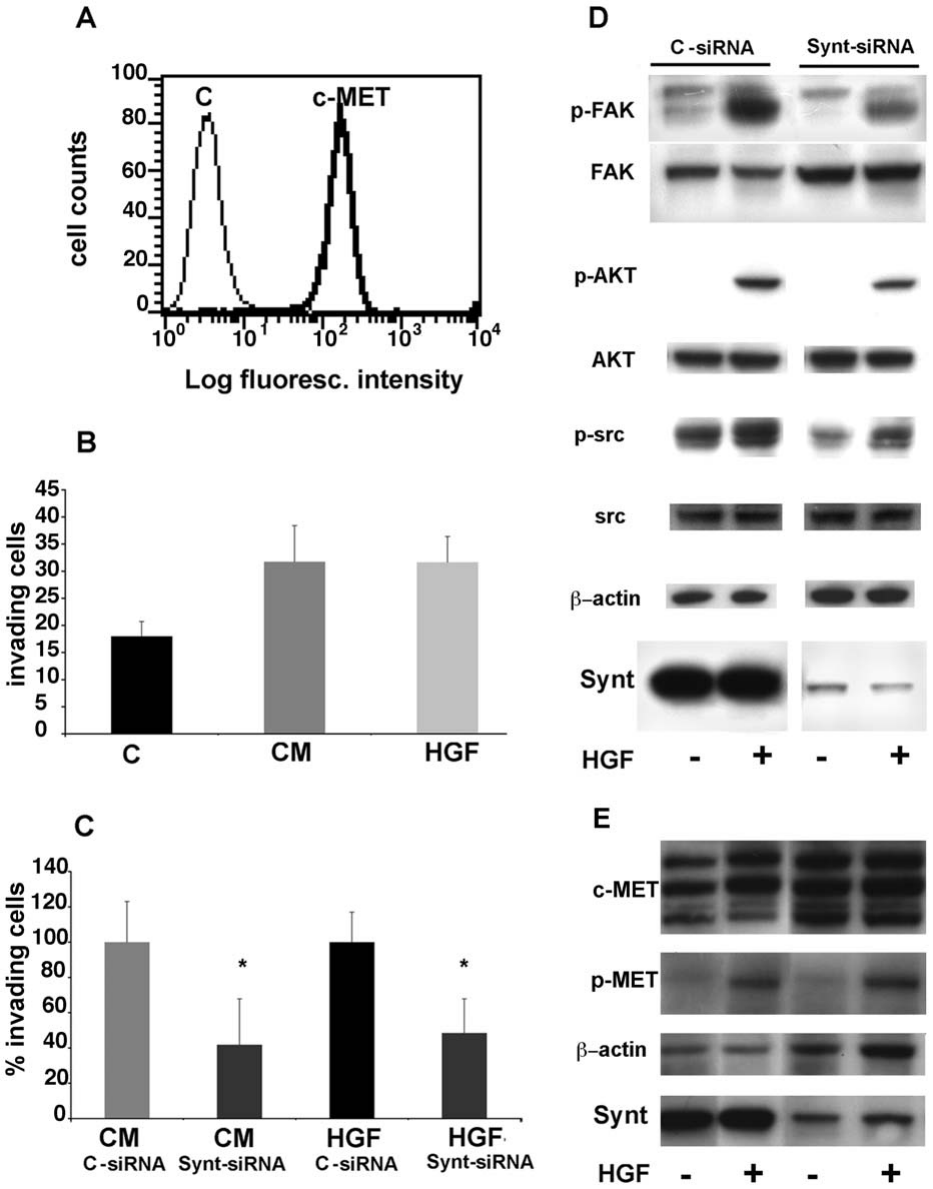
Silencing of mda-9/syntenin in 92.1 uveal melanoma cells inhibits in vitro invasion and HGF mediated signaling. A: Expression of c-MET in 92.1 cells detected by indirect immunofluorescence and flow-cytometry; c: negative control. B: Invasion of matrigel membranes by 92.1 cells towards different stimuli: medium with 10% serum (C), 50% conditioned medium from MG63 cell line (CM), 100 ng/ml recombinant HGF in 0.1% serum. C: Silencing of *SDCBP* (Synt-siRNA) in 92.1 cells inhibits their ability to invade matrigel membranes in response to conditioned medium from MG63 cell line (CM) or recombinant HGF (100 ng/ml). Data are presented as percentage of invading 92.1 cells treated with scrambled siRNA (C-siRNA). * p<0.04. D: Western blot showing inhibition of FAK, AKT and Src phosphorylation in *SDCBP*-silenced 92.1 cells compared to cells treated with scrambled siRNA. The same membrane was also stained for unphosphorylated FAK, AKT and Src , mda-9/syntenin and β-actin as protein loading control. E: Silencing of mda-9/syntenin in 92.1 uveal melanoma cells does not effect c-MET expression and p-MET phosphorylation. Western blot analysis of c-MET, p-MET, mda-9/syntenin and and β-actin as protein loading control in in 92.1 *SDCBP* silenced cells and control siRNA.

We further studied the role of mda-9/syntenin-1 using an invasion assay based on a transwell device in which the two chambers are separated by a matrigel-coated porous set. The MG63 cell conditioned medium, which contains HGF [38], or recombinant HGF were used as stimulus, as HGF has been involved in uveal melanoma migration or invasion [5]–[7]. The 92.1 cell line, which expresses high levels of mda-9/syntenin and the HGF receptor c-MET (Figure 8A) invaded the matrigel membrane in response to MG63 conditioned medium or to recombinant HGF (Figure 8B). *SDCBP* silencing significantly inhibited invasion triggered by both stimuli (Figure 8C). These results indicate that mda-9/syntenin is involved in uveal melanoma cell migration and in their invasiveness triggered by HGF stimulation.

Mda-9/syntenin is known to promote cell motility and invasion by connecting surface integrin signals to FAK activity [28]. In addition, signaling via c-MET is known to activate FAK activity [39], although a role of mda-9/syntenin in this pathway has not been established. We therefore studied the influence of mda-9/syntenin silencing on FAK phosphorylation in response to recombinant HGF. Treatment of 92.1 cell with recombinant HGF for 10 min clearly increased FAK phosphorylation at Tyr397, while total FAK levels were unchanged (Fig. 8D). Silencing the expression of *SDCBP* by siRNA strongly inhibited constitutive and HGF induced Fak phosphorylation (45 and 50% respectively) (Fig. 8D). In addition, mda-9/syntenin silencing also partially inhibited constitutive and/or HGF-promoted Src (15 and 30% respectively) and AKT phosphorylation (20%) in 92.1 cells (Fig. 8D) without affecting neither c-MET expression nor its phosphorylation (Fig. 8E).

### Overexpression of mda-9/syntenin-1 increases invasiveness

**Figure 9:**
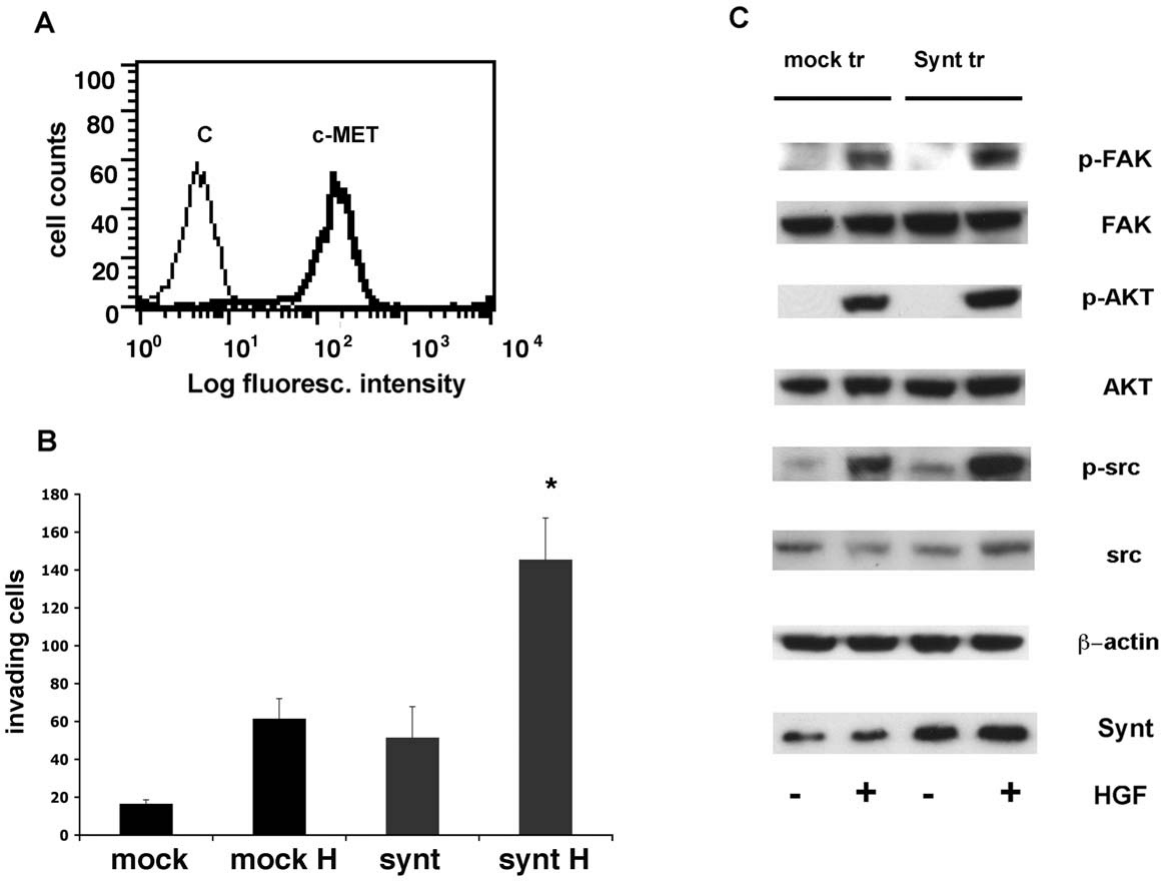
Overexpression of mda-9/syntenin in Mel 270 uveal melanoma cells increases HGF-mediated signaling and invasiveness. A: Expression of c-MET in Mel 270 cells detected by indirect immunofluorescence and flow-cytometry; c: negative control. B: Transfection of *SDCBP* (SDCBP+) in Mel 270 cells enhances their ability to invade matrigel membranes in response to recombinant HGF (100 ng/ml). Data are presented as number of invading cells transfected with mda-9/syntenin vector or empty vector (mock). *: statistically significant difference between HGF induced invasion of mda-9/syntenin and mock transfected cells, p<0.04. C: Western blot analysis of FAK, AKT and Src phosphorylation in *SDCBP*-transfected Mel 270 cells compared to mock-transfected cells. The same membrane was also stained for unphosphorylated FAK, AKT and Src, mda-9/syntenin and β-actin protein as loading control. The second lane was cropped and repositioned.

We further studied the effects of mda-9/syntenin overexepression in gain-of-function studies through *SDCBP* gene transfection in the low-expressing Mel 270 cell line. Also this cell line expressed c-MET (Fig. 9A). *SDCBP*-transfected Mel 270 expressed approximately 40% higher mda-9/syntenin levels (Fig. 9C) and showed increased invasiveness in response to HGF across matrigel-coated porous membranes (Fig. 9B) compared to mock-transfected cells (p<0.04). In addition, mda-9/syntenin overexpression in Mel 270 cells resulted in increased FAK (20%), and Src (30%) phosphorylation in response to HGF stimulation (Fig. 9C), while the effect on AKT activation was modest.

Altogether, loss- and gain-of-function studies suggest that mda-9/syntenin is involved in the activation of an invasive program mediated by HGF in uveal melanoma cells.

## Discussion

Our results provide the first evidence that mda-9/syntenin is expressed in human uveal melanoma and that high level of expression of mda-9/syntenin conferres a high risk of metastatic recurrence. In addition, our present study suggests a role of mda-9/syntenin in promoting metastatic spreading in this tumor. Recent findings have demonstrated that the high expression of mda-9/syntenin is related to the metastatic potential of breast and gastric cancer [27] and cutaneous melanoma cells [25]. The possible role of mda-9/syntenin expression and metastatic progression was demonstrated in cutaneous melanoma, where mda-9/syntenin, through interaction with c-Src/FAK, activates the p38 MAPK/NFkB pathway with subsequent induction of genes involved in migration and invasion [28]. In the present study, a correlation of high *SDCBP* gene expression with metastatic progression was suggested by the analysis of the gene expression profile of 29 primary uveal melanomas. Indeed we found that high *SDCBP* expression conferred a significantly increased risk of metastatic recurrence (Odds ratio of 11.70 p<0,005) in our cohort. The correlation of *SDCBP* mRNA levels with recurrence was further confirmed by the analysis of the raw data of two previously reported [13], [14], [35] datasets of 27 and 63 primary uveal melanomas, respectively. Interestingly, we observed that high expression of *SDCBP* is related to the class 2-gene signature, which has been associated with metastatic behavior of uveal melanoma. It is of note that the top discriminating genes in the previously reported signature were down-regulated genes, whereas *SDCBP* is up-regulated in progressing tumors. Although *SDCBP* maps to 8q12.1, we could not find a significant correlation between chromosome 8 amplification and *SDCBP* gene or protein expression. This may be due either to a partial amplification of *SDCBP* below resolution limit of FISH, or to other mechanisms up-regulating *SDCBP* transcription or mRNA stability. Interestingly, expression of the *PTP4A3* gene mapping on chromosome 8 (8q24.3), has been recently related to uveal melanoma metastatic behavior, and also in this case no correlation with chromosome 8 amplification was found [35].

The immunohistochemical analysis of archival tumors of 29 patients showed expression of mda-9/syntenin protein in all samples, and also higher expression in nine out of eleven metastatic patients analyzed. It is of note that a high level of mda-9/syntenin protein in primary tumors was significantly related to earlier metastatic progression although, further studies involving larger groups of patients are needed to confirm this possibility. Thus, mda-9/syntenin protein, which can be easily detected by immunohistochemistry, correlates with metastatic risk as strongly as monosomy 3 and may represent a candidate prognostic marker of uveal melanoma. Besides the value of mda-9/syntenin expression as prognostic marker in primary tumors, immunohistochemistry of three liver metastases of uveal melanoma showed a very strong mda-9/syntenin staining, suggesting a role of mda-9/syntenin in the metastatic process.

Intriguingly, we also found that mda-9/syntenin is localized not only in the cytoplasm but also in the nuclei of uveal melanoma cells of cell lines or tumor specimen. A noticeable heterogeneity of nuclear localization was observed in different samples. When the intensity of mda-9/syntenin-positive nuclei was considered, no significant relationship with clinical course was evident. Though the possible role of mda-9/syntenin in nuclear functions has yet to be determined in uveal melanoma, recent findings indicated that mda-9/syntenin colocalizes with the SOX-4 transcription factor in the nucleus and stabilizes its expression in different tumor cells [40].

To further study the role of *SDCBP* in uveal melanoma metastases, we developed a pseudometastatic model obtained by intrasplenic injection of uveal melanoma cell lines. The NOD/SCIDIL2Rγ null mice, which in addition to the other immune defects of NOD-SCID mice are also deprived of NK cells [37] allowed a more rapid development of liver metastases than nude and NOD-SCID mice. This finding suggests that NK cells present in the latter strains may partially counteract metastatic dissemination to the liver, suggesting that not only human [36] but also mouse NK cells are able to recognize human uveal melanoma cells. Moreover, our in vivo model of pseudo-metastatic tumor showed a higher expression of mda-9/syntenin in the liver metastases as compared to the spleen, the primary site of injection. A possible explanation of this finding is that tumor cells with the highest expression of mda-9/syntenin are more prone to migrate from the primary tumor and subsequently metastasize. This hypothesis would corroborate the finding of a worse prognosis for those patients expressing high levels of mda-9/syntenin in the primary tumor. Alternatively the liver microenvironment could stimulate mda-9/syntenin expression on the metastatic cells. However, incubation of human uveal melanoma cell lines with mouse liver extracts did not increase mda-9/syntenin expression (data not shown) suggesting that high mda-9/syntenin expressing cells are more prone to metastasize. In addition, our present observation that silencing of *SDCBP* by siRNA inhibits migration and invasiveness of uveal melanoma cells, suggests that mda-9/syntenin is involved in the metastatic dissemination. In this context HGF and its receptor c-MET have been involved in tumor invasiveness and metastatic progression in different types of tumors [41], also including uveal melanoma [5]–[7]. A previous report indicated that HGF enhances migration of uveal melanoma cells [42] and our present study also indicate that the HGF/c-MET axis plays a role in driving invasion. In addition, our data indicate for the first time that mda-9/syntenin is involved in c-MET triggering of invasion, as suggested by *SDCBP* silencing and gain-of-function experiments. Regarding the molecular mechanisms involved, we found that inhibition of mda-9/syntenin expression reduces the activation of FAK, Src and AKT mediated by HGF, whereas its overexpression has opposite effects. Previous data indicated that, upon activation, c-MET can physically interact with FAK, which is an essential kinase involved in the acquisition of an invasive potential [39]. In addition, Mda-9/syntenin has been involved in FAK activation by signals through fibronectin-binding integrins through Src/FAK clustering in cutaneous melanoma [28]. Further studies will be required to establish whether similar molecular clustering mediated by mda-9/syntenin could be involved in c-MET signaling. In conclusion, our present data indicate that *SDCBP* mRNA and mda-9/syntenin protein deserve further investigation as candidate prognostic markers of uveal melanoma and as potential targets for novel therapies aimed at blocking the metastatic process in this tumor.

## Supporting Information

Figure S1 **Nuclear and cytoplasmic localization of mda-9/syntenin in uveal melanoma cell lines.** Confocal microscopy of 92.1 and OMM2.5 cells stained for anti- mda-9/syntenin (green). Nuclei are stained with propidium iodide (red). Z-Y and Z-X sections through a 3-dimensional stack of confocal images show nuclear localization of mda-9/syntenin.
(TIF)

Figure S2 **Nuclear and cytoplasmic localization of mda-9/syntenin in primary uveal melanomas.** Immunohystochemistry of primary uveal melanoma specimens for mda-9/syntenin showed prevalent nuclear (panel A) or cytoplasmic localization (panel B) in different primary uveal melanomas.
(TIF)

Figure S3 **Liver metastases develop earlier in NOD/SCIDIL2Rγ null mice (NOG) (upper panels), than in nude mice (lower panels). **NOG and NU/NU mice were imaged with IVIS imaging system at different time points (15, 22 and 30 days) following spleen transplantation of 10^5^ 92.1 or Mel 270 transduced with a retroviral vector containing the luciferase gene. The signal intensity in the region of interest (ROI) is shown in each mouse.
(TIF)
