# Supplementary figures and images for: Mda-9/Syntenin Is Expressed in Uveal Melanoma and Correlates with Metastatic Progression
Source: PLoS One. 2012 Jan 13;7(1):e29989. doi: 10.1371/journal.pone.0029989 (PMC3258266; doi:10.1371/journal.pone.0029989)

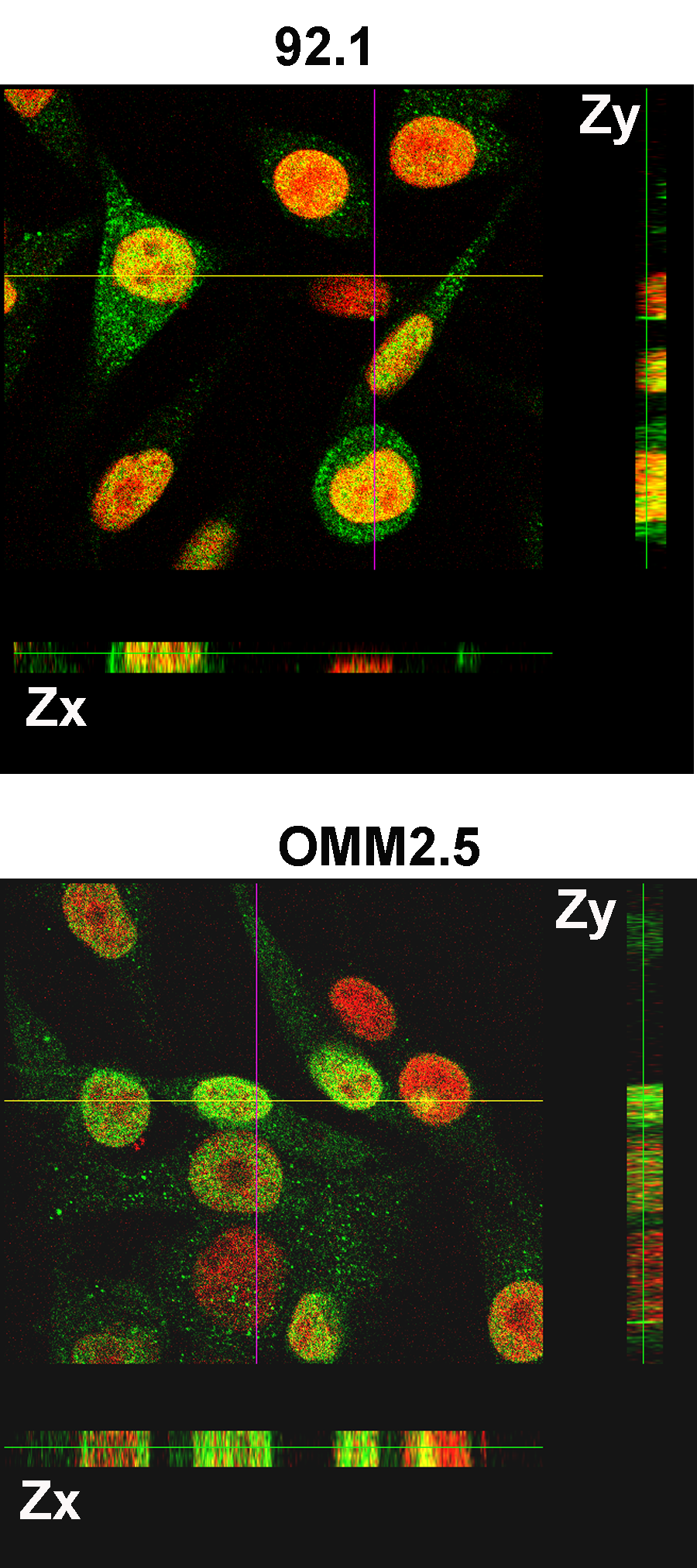

Supplement: Figure S1 — Nuclear and cytoplasmic localization of mda-9/syntenin in uveal melanoma cell lines. Confocal microscopy of 92.1 and OMM2.5 cells stained for anti- mda-9/syntenin (green). Nuclei are stained with propidium iodide (red). Z-Y and Z-X sections through a 3-dimensional stack of confocal images show nuclear localization of mda-9/syntenin. (TIF) [file pone.0029989.s001.tif]

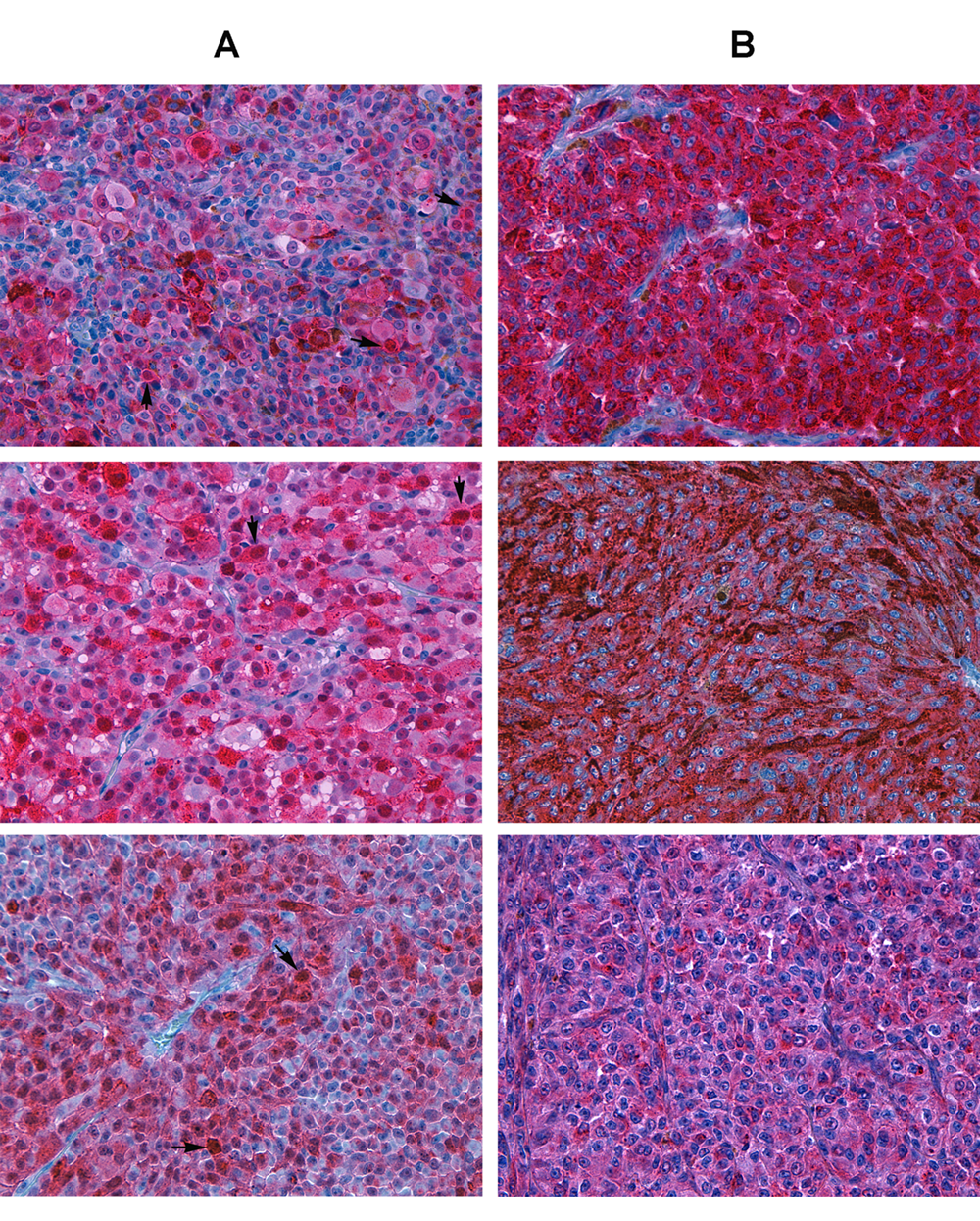

Supplement: Figure S2 — Nuclear and cytoplasmic localization of mda-9/syntenin in primary uveal melanomas. Immunohystochemistry of primary uveal melanoma specimens for mda-9/syntenin showed prevalent nuclear (panel A) or cytoplasmic localization (panel B) in different primary uveal melanomas. (TIF) [file pone.0029989.s002.tif]

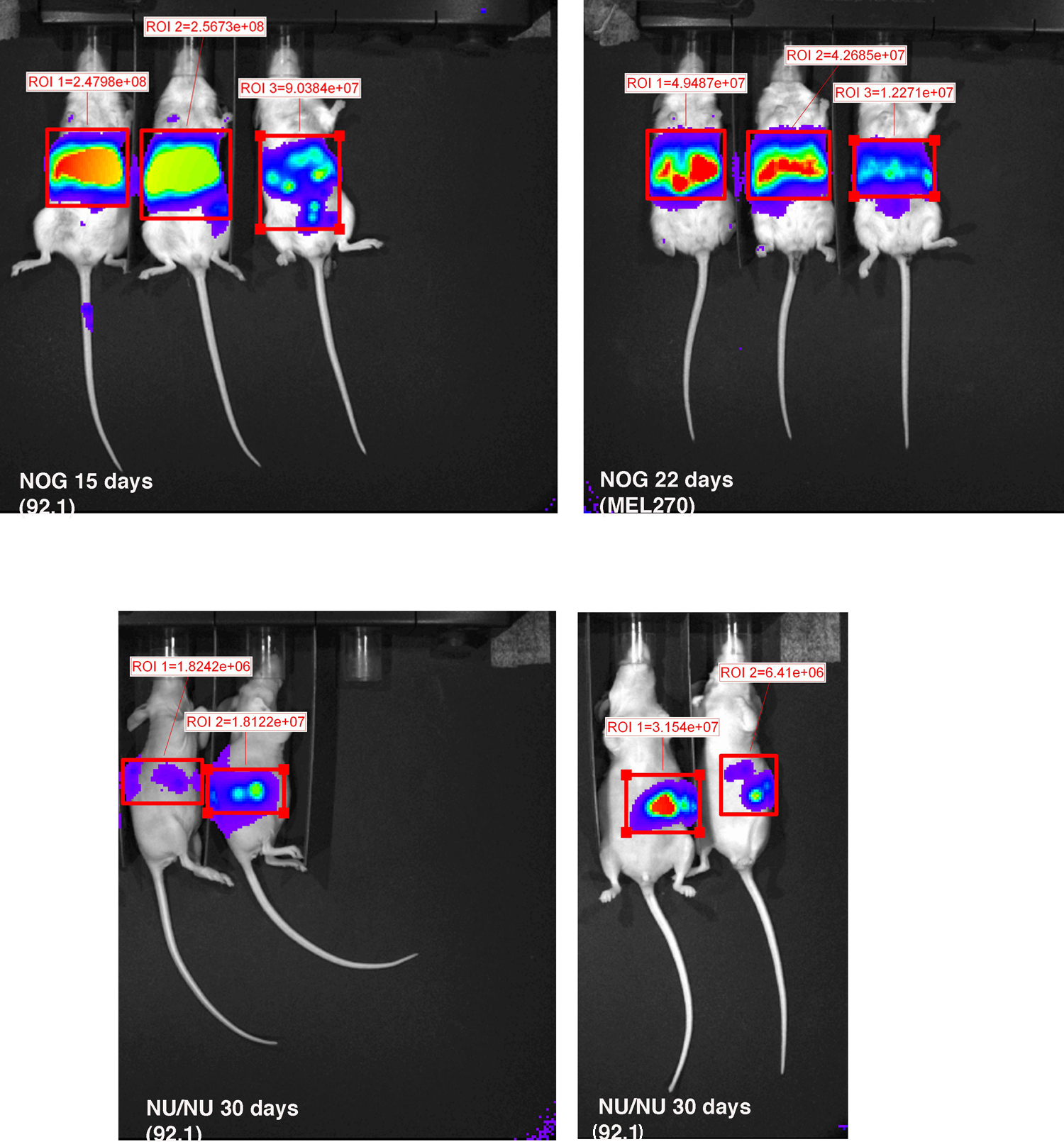

Supplement: Figure S3 — Liver metastases develop earlier in NOD/SCIDIL2Rγ null mice (NOG) (upper panels), than in nude mice (lower panels). NOG and NU/NU mice were imaged with IVIS imaging system at different time points (15, 22 and 30 days) following spleen transplantation of 105 92.1 or Mel 270 transduced with a retroviral vector containing the luciferase gene. The signal intensity in the region of interest (ROI) is shown in each mouse. (TIF) [file pone.0029989.s003.tif]
